# Is there an Association between Oral Health Status and School Performance? A Preliminary Study

**DOI:** 10.5005/jp-journals-10005-1150

**Published:** 2012-08-08

**Authors:** Nishita Garg, Latha Anandakrishna, Prakash Chandra

**Affiliations:** Lecturer, Department of Pedodontics, Sardar Patel Post Graduate Institute of Dental and Medical Sciences, Lucknow, Uttar Pradesh, India e-mail: drnishita_garg@yahoo.com; Professor, Department of Pedodontics, MS Ramaiah Dental College Bengaluru, Karnataka, India; Professor and Head, Department of Pedodontics, MS Ramaiah Dental College, Bengaluru, Karnataka, India

**Keywords:** Oral health status, Academic performance, Caries index

## Abstract

The present cross-sectional study was carried out to assess the impact of poor oral health status on school performance of 600 primary and nursery school children of Bengaluru city, India. The data were collected using the methods and standards recommended by the WHO for oral health surveys. Oral health status was assessed using the df-t index (number of decayed and filled teeth). Academic performance was assessed based on the marks obtained. The children were divided into three groups: Excellent, average, below average (as given by the school teachers). Comparison between categorical variables was performed using one-way ANOVA using the SPSS software package (version 12.0). The mean df-t of the excellent group was 1.56 ± 2.5, for average group it was 2.05 ± 2.8 and for the below average group it was 4.47 ± 2.7. The below average group showed high caries index compared to other groups. The relation between school performance and mean df-t was found to be statistically significant (p < 0.001). The findings of this study demonstrate the impact that poor oral health has, on lowering school performance in children. It can be safely concluded that improvement of children's oral health may be a vehicle to improve their educational experience.

**How to cite this article:** Garg N, Anandakrishna L, Chandra P. Is there an Association between Oral Health Status and School Performance? A Preliminary Study. Int J Clin Pediatr Dent 2012; 5(2):132-135.

## INTRODUCTION

Student health is a strong predictor of academic performance. Healthy, happy, active and well-nourished youth are more likely to attend school, be engaged and be ready to learn. Yet many students come to school with one or more health problems that compromise their ability to learn.^1^ Too many children start first grade with a chronic disease that is largely preventable – tooth decay. Dental caries is the most common chronic disease among children – 5 times more common than asthma.^2^ The effect of caries related pain on distraction from learning and school performance, while not generally measured, is significant. An estimated 51 million school hours per year are lost because of dental-related illness.^3^ A child with a dental problem may have anxiety, fatigue, irritability and depression; he/she may withdraw from normal activities.^4,5^ Children distracted by dental pain may be unable to concentrate and learn, complete school work and score well on tests. The daily nourishment that children receive also affects their readiness for school. Dental problems (e.g. pain, infection and teeth missing due to tooth decay) can cause chewing problems which can limit food choices and result in inadequate nutrition.^6^ Nutritional deficiencies hinder children's school performance, reduce their ability to concentrate and perform complex tasks, contribute to behavioral problems and can have detrimental effects on children's cognitive development and on productivity in adulthood.^7,8^ It is therefore appropriate to hypothesize that poor oral health may burden children in achieving academically, affecting their school performance. To test this hypothesis, a null hypothesis that there is no impact of poor oral health status on school performance, while controlling for other health and sociodemographic factors has been proposed.

## MATERIALS AND METHODS

The study was conducted as part of routine dental screening program carried out by MS Ramaiah Dental College and Hospital, Bengaluru in nursery and primary schools of Bengaluru city. Private schools were randomly selected from the list given by Education Department of Bengaluru to get an equal distribution of children by socioeconomic strata and gender. Sampling method used was stratified random sampling. 600 nursery and primary school children selected by simple random sampling were screened from all the selected schools. For this analysis, only children aged less than 5 years were considered. Informed consent was obtained from the school authorities. The parents or guardians of the subjects also provided written, informed consent. Ethical clearance had been obtained from the Ethics Committee of MS Ramaiah Dental College and Hospital. Oral health status was assessed using the df-t index (number of decayed and filled teeth). All examinations were performed by one of two calibrated examiners who were trained in the assessment of the df-t prior to study initiation, utilizing the WHO criteria (1997) for diagnosis of dental caries. The dental examination was noninvasive (mirror, dental probe, cotton roll) and included optimal illumination of the oral cavity. Radiographs were not used to identify carious lesions. Information about academic performance was obtained from school teachers based on the marks obtained. The children were categorized as excellent (>95% marks), average (50-95% marks), below average (<50%). All statistical analyses were performed using SPSS software (version 12.0 for Windows). The frequency of caries between subgroups was compared using the one-way ANOVA. Least significant difference was calculated and analysis of variance was used to compare outcomes in the excellent and average age groups and average and below average groups.

## RESULTS

The examination of primary school children in Bengaluru city included a convenience sample of 600 students. The gender distribution was even, with 52.5% boys and 47.5% girls, and age ranged from 3 to 5 years. Table 1 shows the mean level of caries prevalence (df-t) for the different subgroups. The mean df-t of the excellent group was 1.56 ± 2.5, for average group it was 2.05 ± 2.8 and for the below average group it was 4.47 ± 2.7, clearly demonstrating a decreasing school performance with high caries index. The relation between school performance and mean df-t was found to be statistically significant (p < 0.001). Table 2 displays subgroup analysis comparing the three groups. Relation between excellent and below average group (p < 0.001) and average and below average group (p = 0.001) was found to be statistically significant. No significant difference was found when comparing the excellent and average groups.

## DISCUSSION

The present survey examined the effect of oral health status on the school performance of children. The effect of caries- related pain on distraction from learning and school performance, while not generally measured, is significant. Left untreated, the pain and infection caused by tooth decay can lead to problems in eating, speaking, and learning.^9^ If a child is suffering pain from a dental problem, it may affect the child's school attendance, and mental and social well-being while at school.^10^ When children's acute dental problems are treated and they are not experiencing pain, their learning and school- attendance records improve.^6^ Episodic pain from dental caries is well-established as a constant finding, even from an early age, affecting up to 20% of preschoolers.^11-14^ Parents of children seeking emergency dental care reported that 19% of the children experienced interference with play, 32% with school, 50% with sleeping and 86% with eating.^12^ One cross- sectional study reported that more than one in 10 schoolchildren experienced tooth pain^15^ and a study in Michigan has documented loss of sleep, inability to concentrate in school and absences from school all caused by dental caries-related pain.^16^ Educational achievement measures, such as school performance are good proxy measures of other key determinants of oral health (e.g. parental education) and are theoretically distinct. In a study done to assess the feasibility and predictive ability of readily available educational indicators as proxy measures of school dental treatment needs in York Region elementary school children, school performance results were found to be good predictors of urgent dental treatment.^17^ In a study done to investigate whether measures of school performance and socioeconomic circumstances could be used as indicators of caries experience in 5-year-old Wandsworth state primary schoolchildren early school performance results in English, mathematics were good indicators of school mean DMFT scores.^18^ Our findings are suggestive of the impact that poor oral health has, on lowering school performance in children. Children in the below average group had significantly higher df-t scores when compared to the average and excellent school performance groups. Our results are in accordance with a study done to examine the sociodemographic and health factors associated with poor school performance among North Carolina children; and the impact of poor oral health status on school performance while controlling for other health and sociodemographic factors.^19^ On analysis it was revealed that sex, race, parental education, low socioeconomic status, poor general health, poor oral health, and the interaction of poor oral health and general health were significantly related to school performance. Children with both poor oral health and general health were 2.3 times more likely to report poor school performance. Children with either poor oral health or general health were only 1.4 times more likely to report poor school performance. Although not all untreated dental caries affects general health, it significantly impacts on the quality of life of children and their dietary intake. In a pilot study assessing the possible effects of extensive dental caries on the quality of life in young children, children with untreated early childhood caries had significantly poorer oral health-related quality of life (OHRQoL) than children without ECC as assessed both by the children and their parents.^20,21^ The consequences of high caries levels also include a higher risk of hospitalizations and emergency dental visits,^22^ increased days with restricted activity and absence from school^3,6,23^ and a diminished ability to learn. Dental pain has an impact not only on the child's educational development, but also on the economy due to time taken off by parents to take children to the dentist.^24^ When considering the results presented, it should be kept in mind that there are some limitations inherent to this study. This study did not control for potential confounders, such as home language and time spent in preschool education, which may affect early literacy. There is no nationally applied test or assessment procedure for this age group. As different education authorities use different accredited baseline testing methods, these results may not apply to schools using alternative tests. The results related to school performance from this study cannot be therefore generalized. Another limitation of this study was its design, which may have underestimated caries experience because of the lack of intraoral radiographs. Our findings support that a healthy child can be expected to perform better in school and add weight to the adage that ‘you can not be healthy without good oral health'. Not withstanding the fact that the results of this preliminary study support an association between oral health status and school performance, future larger survey should incorporate other dental problems, socioeconomic status, an annual estimation of the number of school days missed because of poor oral health and other factors that may act as confounders or effect modifiers. It is important that the health professionals who provide care to children know that oral health cannot be seen as separate from general health and events that take place during childhood can have an impact on adulthood, influencing the child's future health status. Efforts must be made to encourage parents and children to promote and improve their oral health for a better educational experience.

**Table 1:** **Table 1:** Relation between academic performance and df-t

*Groups*		*N*		*Mean df-t*		*SD*		*Min*		*Max*		*F-value*		*p-value*	
Excellent		229		1.56		2.549		0		11		8.245		<0.001	
Average		156		2.05		2.762		0		12					
Below average		215		4.47		2.696		0		10					

SD: Standard deviation

**Table 2:** **Table 2:** Comparison of the three academic groups in relation to df-t

*df-t* *Least significance difference*									
*Performance (I)*		*Performance (J)*		*Mean difference (I-J)*		*Std error*		*Sig*	
Excellent		Average		–0.489		0.347		0.160	
		Below average		–2.909		0.723		<0.001	
Average		Below average		–2.419		0.731		0.001	

## References

[1] Critical connection between student health and academic achievement: How schools and policymakers can achieve a positive impact.. A brief developed jointly by wested and the Philip R. Lee Institute For Health Policy Studies, University Of California, San Francisco On Behalf Of The California Education Supports Project..

[2] Available from: (1996). NCHS, NHANES III [Internet].

[3] Gift HC, Slade GD (1997). Oral health outcomes research: Challenges and opportunities.. Measuring oral health and quality of life.

[4] Ramage S (2000). The impact of dental disease on school performance: The view of the school nurse.. J Southeastern Society Pediatr Dent.

[5] Schechter N (2000). The impact of acute and chronic dental pain on child development.. J Southeastern Society Pediatr Dent.

[6] Reisine ST (1985). Dental health and public policy: The social impact of dental disease.. Am J Public Health.

[7] Available from: Oral health and quality of life. Centers for Disease Control and Prevention [Internet]. 2000.

[8] Rampersaud GC, Pereira MA, Girard BL, Adams J, Metzl JD, (Food Science and Human Nutrition Department, Institute of Food and Agricultural Sciences, University of Florida, SW 23rd Drive, FETL Building 685, Gainesville, FL 32611-0720, USA. gcr@ifas.ufl.edu) (2005). Breakfast habits, nutritional status, body weight, and academic performance in children and adolescents.. J Am Diet Assoc.

[9] Southward LH, Robertson A, Wells-Parker E, Eklund NP, Silberman SL, Crall JJ, Edelstein BL, Baggett DH, Parrish DR, Hanna H, (Social Science Research Center, P.O. Box 5287, Mississippi State, MS 39762, USA. linda.southward@ssrc. msstate.edu) (2006). Oral health status of Mississippi Delta 3- to 5-year- olds in child care: An exploratory study of dental health status and risk factors for dental disease and treatment needs.. J Public Health Dent.

[10] Peterson J, Niessen L, Nana Lopez GM, (Dept. of Biomedical Sciences, Baylor College of Dentistry, Dallas, TX 75266-0677, USA. jpeterson@tambcd. edu) (1999). Texas public school nurses' assessment of children's oral health status.. J Sch Health.

[11] Edelstein B, Vargas CM, Candelaria D, Vemuri M, (Social and Behavioral Sciences, College of Dental Medicine, Columbia University, New York, NY, USA. ble22@ columbia.edu) (2006). Experience and policy implications of children presenting with dental emergencies to US pediatric dentistry training programs..

[12] Clarke M, Locker D, Berall G, Pencharz P, Kenny DJ, Judd P, (Community Dental Services Research Unit, Faculty of Dentistry, University of Toronto, Ontario, Canada. martha.clarke@utoronto.ca) (2006). Malnourishment in a population of young children with severe early childhood caries.. Pediatr Dent.

[13] Tickle M, Blinkhorn AS, Milsom KM, (School of Dentistry, University of Manchester, Manchester, UK. martin.tickle@manchester.ac.uk) (2008). The occurrence of dental pain and extractions over a 3-year period in a cohort of children aged 3-6 years.. J Public Health Dent.

[14] Siegal MD, Yeager MS, Davis AM, (Bureau of Oral Health Services, Ohio Department of Health, Columbus, Ohio, USA. msiegal@odh.ohio.gov) (2004). Oral health status and access to dental care for Ohio Head Start children.. Pediatr Dent.

[15] Meadow D, Edelstein B (1981). An evaluation of the management of dental emergencies by the school nurse.. Pediatr Dent.

[16] Available from: Children's oral health: More vigilance needed–study shows effects on quality of life. Dental UM [Internet]. 2006 [cited 2006]. “www.dentumich.edu/alumni/dentalum/springsummer2006/Childrens_Oral_Health.pdf”..

[17] Muirhead VE, Locker D, (Faculty of Dentistry, University of Toronto, 124 Edward Street, Toronto, Ontario, Canada, M5G 1G6. vanessa.muirhead@utoronto.ca) (2006). School performance indicators as proxy measures of school dental treatment needs: A feasibility study.. J Public Health Dent.

[18] Muirhead V, Marcenes W, (Community Dental Service, Wandsworth Primary Care Trust, London. vanessa.muirhead@utoronto.ca) (2004). An ecological study of caries experience, school performance and material deprivation in 5-year-old state primary school children.. Community Dent Oral Epidemiol.

[19] Blumenshine SL, Vann WF Jr, Gizlice Z, Lee JY, (Department of Pediatric Dentistry, University of North Carolina, Chapel Hill, NC 27599, USA. blumenss@dentistry.unc.edu) (2008). Children's school performance: Impact of general and oral health.. J Public Health Dent.

[20] Low W, Tan S, Schwartz S, (Pediatric Dentistry, McGill University, Montreal) (1999). The effect of severe caries on the quality of life in young children.. Pediatr Dent.

[21] Filstrup SL, Briskie D, da Fonseca M, Lawrence L, Wandera A, Inglehart MR, (School of Dentistry, University of Michigan, Ann Arbor, Mich, USA. sarafio@msn.com) (2003). Early childhood caries and quality of life: Child and parent perspectives.. Pediatr Dent.

[22] Majewski RF, Snyder CW, Bernat JE, (Children's Hospital of Buffalo) (1988). Dental emergencies presenting to a children's hospital.. ASDC J Dent Child.

[23] Pongpichit B, Sheiham A, Pikhart H, Tsakos G, (Sirindhorn College of Public Health, Muang District, Khon-Kaen, Thailand. pbussayasit@yahoo.com) (2008). Time absent from school due to dental conditions and dental care in Thai schoolchildren.. J Public Health Dent.

[24] Sheiham A, (Department of Epidemiology and Public Health, University College London, 1-19 Torrington Place, London, WC1E 6BT. a.sheiham@ucl.ac.uk). (2006). Dental caries affects body weight, growth and quality of life in pre-school children.. Br Dent J.

